# Multidrug Resistance in Breast Cancer: From *In Vitro* Models to Clinical Studies

**DOI:** 10.4061/2011/967419

**Published:** 2011-02-24

**Authors:** N. S. Wind, I. Holen

**Affiliations:** Academic Unit of Clinical Oncology, DU10, Medical School, University of Sheffield, Beech Hill Road, Sheffield S10 2RX, UK

## Abstract

The development of multidrug resistance (MDR) and subsequent relapse on therapy is a widespread problem in breast cancer, but our understanding of the underlying molecular mechanisms is incomplete. Numerous studies have aimed to establish the role of drug transporter pumps in MDR and to link their expression to response to chemotherapy. The ATP-binding cassette (ABC) transporters are central to breast cancer MDR, and increases in ABC expression levels have been shown to correlate with decreases in response to various chemotherapy drugs and a reduction in overall survival. But as there is a large degree of redundancy between different ABC transporters, this correlation has not been seen in all studies. This paper provides an introduction to the key molecules associated with breast cancer MDR and summarises evidence of their potential roles reported from model systems and clinical studies. We provide possible explanations for why despite several decades of research, the precise role of ABC transporters in breast cancer MDR remains elusive.

## 1. Introduction

Resistance to chemotherapy is a major problem in the management of breast cancer, where many of the initially responsive tumours relapse and develop resistance to multiple anticancer agents of different structure and mechanism of action [1]. This phenomenon is known as multidrug resistance (MDR). The precise nature of chemotherapy resistance, and the potential role of drug resistance genes involved in the transport of anticancer agents, is still unclear. A better understanding of the underlying molecular mechanisms of chemotherapy resistance is required in order to develop successful therapeutic strategies to overcome MDR.

 Drug resistance can be mediated by a number of different mechanisms. It may be due to an increase in the activity of ATP-dependent efflux pumps resulting in reduced intracellular drug concentrations. Agents commonly associated with this type of resistance include doxorubicin, daunorubicin, vinblastine, vincristine and paclitaxel [2]. It can also be caused by a reduction of cellular drug uptake. Water-soluble drugs may attach to transporters carrying nutrients and therefore fail to accumulate within the cell. Resistance to drugs like cisplatin, 8-azaguanine and 5-fluorouracil is mediated by this mechanism [3]. Another general mechanism of resistance involves the activation of regulated detoxifying systems such as the cytochrome P450 mixed function oxidases, and also of increased DNA repair. In addition, resistance can result from defective apoptotic pathways due to malignant transformation [4], a change in the apoptotic pathway during exposure to chemotherapy [5], or changes in the cell cycle mechanisms that activate checkpoints and prevent initiation of apoptosis. Other mechanisms involved in drug resistance include lack of drug penetration, modification of the ability to activate prodrugs and alterations in drug targets. This paper will describe the main molecules and mechanisms involved in MDR in breast cancer, and summarise the results from key *in vitro*, *in vivo* and clinical studies investigating their respective roles.

## 2. ABC Transporters

Several transmembrane transporter proteins have been shown to be involved in the resistance of tumour cells to chemotherapeutic agents. These proteins are termed ATP-binding cassette transporters (ABC-transporters), and utilise the energy of adenosine triphosphate (ATP) hydrolysis to carry out biological processes. ABC transporters can be divided into three functional categories: *Importers* mediate the uptake of nutrients into the cell (amino acids, sugars, ions and other hydrophilic molecules). *Exporters/effluxers* pump toxins and drugs out of the cell. The final category of ABC proteins are involved in *translation and DNA repair processes*. 49 human ABC genes have been identified to date, these have been divided into 7 subfamilies (ABCA-ABCG) based on their sequence homology and domain organisation [14].

**Figure 1:**
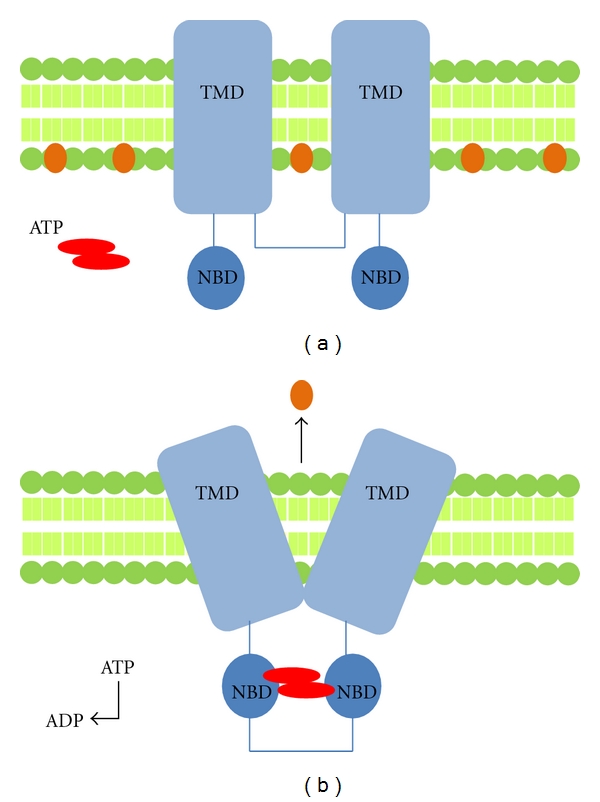
(a). An example of the general structure an ABC transporter with 2 sets of transmembrane domains (TMD) and 2 nucleotide binding domains (NBD). Substrate molecules are present in the inner membrane shown in orange. Upon binding of ATP, the NBD become joined, leading to a conformational change (b). This change causes the movement of the substrate out of the membrane.

All proteins in the ABC family are characterised by two distinct domains, the transmembrane domain TMD (also known as the membrane spanning domain or the integral membrane domain) and the nucleotide-binding domain (NBD) (Figure 1). The TMD recognizes a variety of substrates and undergoes conformational changes to transport these across the membrane. The sequence and structure of TMDs is variable, reflecting the chemical diversity of substrates that can be translocated. The NBD or ATP-binding cassette (ABC) domain is located in the cytoplasm and has a fixed sequence and structure where ATP-binding occurs [15].  Table 1 gives an overview of a number of different ABC transporters that have been linked to MDR in breast cancer.

We will focus on P-glycoprotein (PGP), multidrug resistance-associated protein 1 (MRP1) and breast cancer resistance protein (BCRP), the main ABC transporters implicated in the development of multidrug resistance in breast cancer.

## 3. P-glycoprotein (PGP)

**Figure 2:**
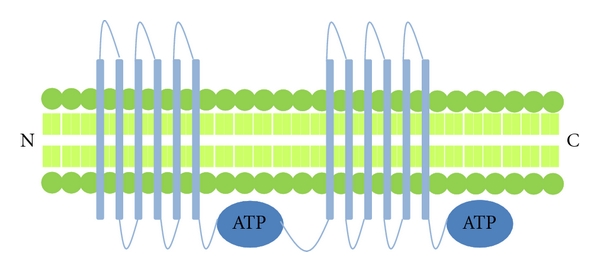
Structure of P-glycoprotein (PGP)—this ABC transporter consists of 12 transmembrane domains and 2 ATP binding sites. Other transporters with a similar structure include MDR4, MRP4, MRP5 and MRP7.

P-glycoprotein has a wide tissue distribution [16] and was the first ABC transporter identified to be overexpressed in breast cancer cell lines displaying MDR [17]. Mouse PGP, which has 87% sequence morphology to human PGP in a drug-binding state, has recently been described [18]. PGP is a broad spectrum multidrug efflux pump that has 12 transmembrane domains and two ATP-binding sites [19] (Figure 2). It is involved in the transport of neutral and cationic hydrophobic compounds (vinblastine, vincristine, doxorubicin, daunorubicin, etoposide and paclitaxel) out of cells. For transport via PGP, extraction of the drug directly from the cytoplasmic side of the lipid bilayer often occurs. Most PGP substrates readily partition into the plasma membrane and lipids are required for drug stimulated ATPase activity. PGP is a unidirectional lipid flippase that transports phospholipids from the inner to outer sections of the bilayer [18].

## 4. MDR-Associated Protein (MRP1)

**Figure 3:**
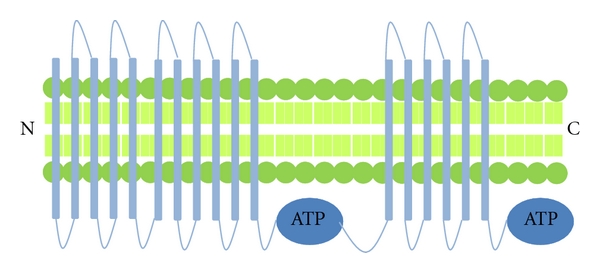
Structure of Multidrug Resistance Protein 1 (MRP1)—this ABC transporter is similar in structure to PGP in that they possess 2 ATP binding sites. In addition to the 12 transmembrane domains, they also contain an additional 5 transmembrane domains at the amino terminal end. Other transporters with a similar structure include MRP2, MRP3 and MRP6.

MRP1 is also expressed in many different organs and cell types, including breast cancer cells [20]. Studies have demonstrated that overexpression of MRP1 leads to cells becoming resistant to a wide variety of anticancer drugs, for example, doxorubicin [21]. MRP1 is a drug efflux transporter with broad substrate specificity. For many drugs, MRP1-mediated transport is stimulated by the presence of glutathione. [22]. Unlike PGP, which tends to be located in the apical membranes of epithelial cells, MRP1 is located basolaterally. MRP1 has a similar structure to PGP, and also requires two molecules of ATP as its energy source, but the nucleotide binding sites 1 and 2 (NBD1 and NBD2) differ in their affinity for ATP (Figure 3). The substrate binds to the MRP1 transmembrane domain causing a conformational change of the protein, which initially induces ATP-binding at NBD1. Further changes in conformation enhance ATP binding at NBD2. When both NBD1 and NBD2 are occupied, the bound substrate is transported out of the cell. ATP bound at NBD2 is then hydrolysed, and the subsequent release of ADP from NBD2 partially returns MRP1 back to its original conformation, facilitating the release of the ATP bound at NBD1 completing the cycle.

## 5. Breast Cancer Resistance Protein (BCRP)

**Figure 4:**
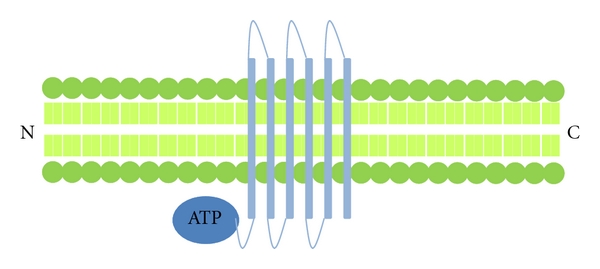
Structure of Breast Cancer Resistance Protein (BCRP)—this ABC transporter contains 6 transmembrane domain and 1 ATP binding site on the amino terminal side of the transmembrane domain. This is known as a “half transporter,” these are thought to form dimmers in order to function.

BCRP is expressed in a variety of tumours and is associated with resistance to a wide range of different anticancer agents including mitoxantrone, camptothecins, anthracyclines, flavopiridol and antifolates [23]. Unlike PGP and MRP1, the BCRP protein contains only one transmembrane domain and one nucleotide binding domain (Figure 4). Two molecules of BCRP are bound by a disulfide bridge to form a functioning homodimer [24]. The mechanism of drug transport facilitated by BCRP has not been investigated in as much detail as that of PGP and MRP1, but the basic steps are thought to be similar, involving a cycle of substrate transport and ATP hydrolysis [16]. Stem cells and tumour cells in a hypoxic environment may be protected from chemotherapeutic agents due to an increased expression of BCRP induced by hypoxia [25]. However, this may not be the case for all stem cells. Hoechst 33342 and rhodamine-123 have been used to investigate the efflux efficiency of these substrates in mammary stem cells [26]. Hoechst 33342 is a substrate of BCRP and causes BCRP-positive cells to display a unique “side population” phenotype [21, 27]. In the work by Stingl et al., a small proportion of mammary stem cells were found to possess a side population phenotype and only a small minority of cells effluxed Hoechst or rhodamine. These data suggest that in contrast to haematopoietic stem cells [26], there is no increase in BCRP in the mammary stem cells.

## 6. Drug Resistance in Breast Cancer

Chemotherapy is central in the treatment of breast cancer, but the development of drug resistance remains a problem. Response rates to first line chemotherapies in metastatic breast cancer, either single or a combination of drugs, are around 30%–70%, and the disease-free period following treatment is often only 7–10 months [28].  Table 2 gives an overview of the main chemotherapy drugs used to treat breast cancer and their mechanism of action. The role of ABC transporters in breast cancer MDR has been investigated by evaluation of gene and protein expression in tumour samples using RT-PCR, Western blot and immunohistochemistry. The levels of expression have then been scored and linked to treatment response and outcome. As will be discussed in more detail in later sections, the data reported from these studies have been conflicting, most likely caused by a number of variable factors. For example, it is difficult to generate accurate overall measures of ABC transporter expression due to the heterogeneity of the tumours and changes in expression due to therapy. In addition, the large number of different proteins involved in mediating MDR means that there is considerable redundancy in the system. Ultimately, it is the combined activity of the expressed ABC transporters over the course of disease progression that determines the tumour response to therapy. This highly dynamic system cannot be adequately captured simply by taking a snapshot of the tumour.

## 7. Multidrug Resistance in Breast Cancer Cells *In Vitro*

As studies in clinical material do not allow experimental manipulation essential for dissecting the complex role of ABC transporters in MDR, researchers have turned to* in vitro* models. Expression levels of the relevant proteins have been modified in breast cancer cell lines, and the resulting changes in sensitivity to various chemotherapeutic agents assessed. Effects of anticancer drugs on expression levels of the individual ABC transporters have also been determined, alongside functional assays of ABC-mediated drug transport across cell monolayers. Here we describe some examples of *in vitro* approaches that have been utilised to investigate the relationship between expression and activity of ABC transporters and sensitivity to chemotherapy agents.

Hembruff and colleagues generated a panel of MCF-7 cell lines selected for resistance to various chemotherapy drugs, and used these to study how expression of drug transporters related to drug uptake and sensitivity [29]. The cell lines were resistant to either paclitaxel (MCF-7_tax-2_), docetaxel (MCF-7_txt_), doxorubicin (MCF-7_dox-2_) or epirubicin (MCF-7_epi_). Cellular uptake of ^3^H-paclitaxel, doxorubicin and epirubicin was evaluated to determine any relationship between drug accumulation and resistance. A threshold drug concentration was required for both taxanes and anthracyclins for the cells to acquire drug resistance, and there was a significant degree of cross-resistance to drugs of the same class. Taxane-resistant cells exposed for 2 weeks to increasing concentrations of taxanes had significantly reduced ^3^H-paclitaxel accumulation, with uptake as low as 2% of control in MCF-7_tax-2_ cells. Very similar data were observed for anthracyclin-resistant cell lines, anthracyclin-resistance was associated with a reduction in drug uptake. However, in both cases there was no clear, dose-dependent correlation between changes in drug accumulation and degree of resistance. Whether the levels of expression of MDR-associated transporters were linked to acquisition of drug resistance was determined by real-time PCR analysis and western blotting. There was a substantial increase in ABCB1/PGP protein levels in MCF-7_tax-2_, MCF-7_txt_, and MCF-7_epi_ and in ABCC1/MRP1 in MCF-7_dox-2_ cells, supporting that drug resistance is associated with both modified drug accumulation and increased levels of a subset of ABC transporter proteins. Taken together, the data in this study suggest that whereas there is a link between the onset of drug resistance and reduced drug uptake, additional mechanisms must be involved in determining the sensitivity of the cells to chemotherapy agents. 

One method for determining the functional activity of ABC transporters is by using the Caco-2 cell model of transepithelial drug transport [30]. For the measurement of apical to basolateral drug transport (i.e., absorptive), the drugs are added to the apical side of the cell monolayer and medium added to the basolateral side. At regular time intervals medium is removed from the basolateral side and the concentration of drug determined using high performance liquid chromatography. The measurement of basolateral to apical drug transport (i.e., secretory) is measured in the same system by adding drugs to the opposite side of the monolayer. When this model system was used to study transport of belotecan and topotecan in the presence of PGP, MRP2 and BCRP inhibitors, the inhibitors caused a significant reduction in the secretory flux of both drugs. Consistent with this decrease, the absorptive fluxes of the drugs were significantly increased by the apical presence of the inhibitors of PGP and MRP1, but not by inhibitors of MRP2 or BCRP. These data suggest that BCRP, PGP and MRP2 are all involved in the transport of belotecan and topotecan, supporting that there is considerable redundancy in the MDR system/components. Other models for investigating transepithelial drug transport include the use of MDCKII and LLC-PK cells overexpressing one or several of the ABC transporters. A difference between efflux ratios in the transfected cells compared to the parental cells lines indicates transporter-mediated active drug uptake or efflux.

The activity of ABC transporters (PGP, MRP1 and BCRP) has also been investigated in MCF-7 wild-type and BCRP overexpressing breast cancer cells [31]. The accumulation of mitoxantrone and pheophorbide A was studied in the presence of 50 *μ*M tetrahydrocurcumin (a metabolite of curcumin). Tetrahydrocurcumin inhibited the efflux of mitoxantrone and pheophorbide A in the BCRP overexpressing cells, but no effect was observed in the wild type cells. The group also assessed the activity of PGP by determining the intracellular retention of [^3^H]-vinblastine in drug resistant MCF-7MDR [32] and sensitive MCF-7 cells. Only in the drug resistant cells did exposure to tetrahydrocurcumin result in a significant dose-dependent increase of [^3^H]-vinblastine accumulation when compared with a dimethyl sulfoxide control. Tetrahydrocurcumin was seen to activate PGP-mediated ATPase and to stimulate ATPase activity of BCRP. [^125^I]-Iodoarylazidoprazosin ([^125^I]-IAAP), a photoactive analogue of prazosin, was used in this study to characterise the drug binding sites of PGP and BCRP. Tetrahydrocurcumin inhibited the incorporation of [^125^I]-IAAP into PGP and BCRP in a dose-dependent manner, suggesting that this drug binds directly to the substrate binding sites of PGP and BCRP. Tetrahydrocurcumin was found to increase etoposide sensitivity in MRP1 overexpressing cells and increase the sensitivity of cells that overexpressed BCRP to mitoxantrone, and sensitised drug resistant cells to vinblastine; suggesting a reversal activity of tetrahydrocurcumin on the PGP-mediated MDR phenotype.

Although ABC transporters are mainly localised in the plasma membrane [33], they are also expressed in subcellular compartments where they actively sequester drugs away from their (cytoplasmic) targets [34]. It has been hypothesised that BCRP could be expressed in the mitochondria and thereby be involved in maintaining low concentrations of anticancer drugs [35]. The functional activity of BCRP has been investigated measuring the uptake of rhodamine 123 (rho 123) and mitoxanatrone in the presence or absence of 10 *μ*mol/L of the BCRP inhibitor Fumitremorgin C [35]. This drug caused increased accumulation of mitoxantrone in parental (drug sensitive) cells but had no effect on drug uptake. Mitoxantrone accumulation in the cell lines that overexpressed BCRP was significantly reduced following exposure to Fumitremorgin C compared with the parental cell lines, possibly due to BCRP-mediated efflux. To establish whether BCRP is functionally active in the mitochondria, efflux experiments were carried out in isolated mitochondria. Mitoxantrone efflux was elevated in mitochondria from the BCRP overexpressing cells compared with levels seen in mitochondria from the parental cells. These data suggest that BCRP is functionally expressed in the mitochondria of MDR cell lines and may be involved in protecting mitochondria DNA from damage by chemotherapy drugs. 

In 2007 a case study was published [36] exposing the misidentification of the cancer cell line MCF-7AdrR (later redesignated NCI/ADR-RES). These cells have been widely used in research into multidrug resistance in breast cancer during the last two decades. This study revealed that these cells are derived from OVCAR-8 ovarian adenocarcinoma cells and are not of breast origin as first thought. The consequences of this misidentification leads to the need for many studies to be reevaluated and relevant conclusions made using OVCAR-8 cells as controls. In terms of breast cancer research the studies would need to be repeated using alternative cell lines.

## 8. Multidrug Resistance in *In Vivo* Models of Breast Cancer

To gain further understanding of the complex system involved in MDR, it is necessary to use *in vivo* models that contain multiple cell types, and that include the tumour vasculature and immune cells. These models can also be used to investigate the effects of therapy on tumours in which MDR-related genes have been either overexpressed or knocked out. Two commonly used models are xenograft implantation of human breast cancer cells in immunocompromised mice, and a genetically engineered mouse model of hereditary breast cancer. The following section will review data reported from studies that have used *in vivo* models to investigate the role of the ABC transporters on treatment response.

A genetically engineered mouse model of hereditary breast cancer (*K14cre; Brca1^F/F^; p53^F/F^*) has recently been used to investigate drug resistance [37]. In this model, the mammary tumours that spontaneously develop mimic key features of human breast cancer-(BRCA1)-associated mammary carcinomas. BRCA1 is essential for the repair of double-stranded DNA breaks by homologous recombination and hence the *Brca1^−/−^/p53^−/−^* tumours tested were sensitive to the DNA interacting drugs cisplatin, doxorubicin, topotecan and carboplatin. The tumours could not be eradicated and eventually acquired resistance to doxorubicin and topotecan. A major characteristic of the doxorubicin resistance was found to be increased expression of *mdr1a* and *mdr1b* genes that encode the murine drug transporter PGP. A 5-fold increase above the average *mdr1a* and *mdr1b* transcription levels of untreated tumours was sufficient to cause doxorubicin resistance [37]. *Mdr1* gene expression levels were determined in doxorubicin-sensitive and doxorubicin-resistant mouse mammary tumours in comparison with selected normal tissue from the large intestine (*mdr1a*) or kidneys (*mdr1b*). The average level of *mdr1* mRNA in untreated tumours was comparable with the normal tissue. In 11 out of 13 resistant tumours at least a 2-fold increase of *mdr*1 mRNA levels above the average of untreated tumours was detected. Doxorubicin-resistant tumours have been shown to largely maintain their resistance phenotype following subsequent orthotopic transplantation into syngeneic animals [38].

The importance of PGP in doxorubicin-resistance has been investigated using the PGP inhibitor tariquidar in *Brca1^−/−^;p53^−/−^* mammary tumours generated in *K14cre;Brca1^−/−^;p53^−/−^* mice [39]. Administration of tariquidar (10 mg/kg) before doxorubicin (5 mg/kg) successfully reversed doxorubicin resistance in three individual tumours which had increased level of *mdr1* mRNA. However, despite the tumours becoming resensitised to doxorubicin they were not eradicated by the treatment combination of doxorubicin and tariquidar. The authors suggest this may be due to dormancy of residual tumour-initiating cells, and that inhibiting PGP is not enough to resensitise all tumour cells to doxorubicin [37]. These results highlight an important limitation of the potential use of PGP inhibitors in the clinical setting.

PGP has also been shown to contribute to resistance of novel targeted therapies such as the poly-(ADP-ribose) polymerase 1 (PARP 1) inhibitor AZD2281 [40]. In a genetically engineered mouse model (GEMM) for BRCA1-associated breast cancer, AZD2281 treatment inhibited tumour growth without signs of toxicity. However, long-term treatment resulted in the development of drug resistance which was associated with an up-regulation of genes encoding for the PGP efflux pump. This resistance to AZD2281 was reversed by coadministration of the PGP inhibitor tariquidar, supporting that the resistance was mediated through increased PGP expression levels.

The (*K14cre;Brca1^−/−^;p53^−/−^*) mouse model has also been used to demonstrate the involvement of BCRP in resistance to topotecan. In this study, tumour bearing animals were treated with topotecan, alone or in combination with PARP inhibitor olaparib [41]. Although topotecan treatment did prolong survival, all tumours eventually acquired resistance. This may have been caused by overexpression of BCRP and/or reduced levels of the drug target. Tumour-specific ablation of *Abcg2 *(the gene coding for BCRP) significantly reduced tumour growth and increased overall survival of topotecan treated animals. Despite a lack of BCRP, none of the BCRP*^−/−^*;BRCA1*^−/−^*;p53*^−/−^* tumours were completely eradicated, even in the treatment group including olaparib. However, it was noted that olaparib substantially increased topotecan toxicity, and this may also occur in humans. The study supports that BCRP expression is involved in the development of resistance to topotecan.

As already mentioned, tumour heterogeneity combined with biopsy inaccuracy contributes to variability and limit histological and mRNA based detection of MDR pump proteins [42]. Therefore it would be useful to be able to quantify levels of pump protein expression throughout the whole tumour, rather than taking a local biopsy. Noninvasive imaging of PGP-mediated transport has been developed, using a range of radiolabelled drugs including daunorubicin [43], and metal complexes such as ^99m^Tc-MIBI [44]. Although this is a reproducible technique, the need for specialised equipment precludes routine use. Van Leeuwen and colleagues have demonstrated the potential for functional imaging techniques using the *K14cre; Brca1^F/F^; p53^F/F^* mouse model for hereditary mammary carcinoma [45]. Small pieces of tumours expressing either basal, intermediate, or high levels of *mdr1a/b* (the mouse equivalent of PGP) were implanted in the mammary fat pad of wild type F1 animals. ^99m^Tc-MIBI time intensity curves were generated for each of the tumours during the first 30 minutes after injection. Comparison of the tumours expressing basal and high levels of PGP revealed that elevated PGP expression can be directly correlated with an increase in the ^99m^Tc-MIBI efflux rate. Administration of the PGP inhibitor tariquidar (10 mg/kg, 10 minutes before imaging the tumours) showed that in the presence of the inhibitor the rate of ^99m^Tc-MIBI efflux did not depend on *mdr1a/b* expression levels. These data support that ^99m^Tc-MIBI imaging effectively visualises the effect of PGP inhibitors on PGP-mediated transport. The authors suggest that it is possible to classify tumours based on their PGP transport activity, and also to directly link treatment outcome to the measured PGP-mediated ^99m^Tc-MIBI efflux rates. Their model suggests a high probability of drug resistance in the intermediate and high PGP expression level tumours, demonstrating the potential for future clinical use of functional imaging in predicting MDR.

## 9. Multidrug Resistance in Breast Cancer—Clinical Studies

It is well established that many breast tumours that initially respond to treatment subsequently develop resistance to a broad range of drugs. Currently anthracycline-based chemotherapy is a standard treatment for breast cancer, with doxorubicin and its analogue epirubicin extensively used in combination with 5-fluorouracil and cyclophosphamide. All of these compounds are substrates for the ABC transporters and are therefore subject to MDR. As a result, a number of studies have aimed to relate the levels of MDR-pumps in breast tumours to clinical outcome, and we give some examples in the following sections.

Park and colleagues used gene expression profiling to determine whether the expression pattern of a panel of ABC transporters can be used to predict response to neoadjuvant chemotherapy in breast cancer [46]. 21 patients received 4 courses of 5-fluorouracil, epirubicin and cyclophosphamide, followed by a 12-week-course of paclitaxel, and were then split into two groups; Those that had no pathological evidence of any residual cancer cells and those with some residual tumour after completion of neoadjuvant chemotherapy. The average tumour expression of each transcript on the ABC transporters was determined using PCR. In tumour samples taken before treatment started a number of ABC transporters were expressed at over 50-fold higher levels than the median values. This large variation in expression levels between the individual tumours may have been due to differences in disease stage, human epidermal growth factor status, oestrogen receptor status and/or node status. Following microarray analysis, several of ABC transporters showed differential expression between the two groups of patients. ABCC7, ABCF2 and ABCB2 were expressed at high levels in the tumours of patients with no residual disease. In contrast, ABCC5, ABCA12, ABCA1, ABCC13, ABCB6, and ABCC11 were expressed at significantly higher levels in the patients with residual disease and this was associated with decreased in responsiveness to neoadjuvant therapy. ABCC5 has been reported to confer resistance to 5-fluorouracil [47] and showed the highest gene expression level in tumours with decreased response. The authors suggest that establishing the tumour ABC transporter gene expression profile may be useful in predicting the pathological response to 5-Fluorouracil, epirubicin and cyclophosphamide treatment in breast cancer patients, but a larger cohort of patients needs to be investigated to confirm whether this is the case.

The potential prognostic impact of MDR gene expression in breast cancer patients has also been investigated in the adjuvant setting [48]. Expression of PGP and MRP1 was measured in breast cancer tissue from 171 patients treated by surgery, adjuvant chemotherapy +/− radiotherapy +/− hormonal therapy. Using RT-PCR, 58% of the tumours (*n* = 68) expressed PGP and MRP1 was expressed in 92.4% out of 131 tumours. This study did not reveal any statistically significant correlation between PGP and MRP1 expression and the 5-year disease-free survival or overall survival. 

A number of studies have been designed to demonstrate a link between expression of MDR proteins and response to treatment/survival in breast cancer. In a study of 85 node-positive breast cancer patients receiving anthracycline-based adjuvant therapy, no significant influence of PGP or MRP1 expression was seen on progression-free or overall specific survival [49]. This study was supported by data observed by Kanzaki et al. [50]. Expression levels of PGP, MRP1 and BCRP were measured using RT-PCR in tumours from 38 breast cancer patients that received doxorubicin-based chemotherapy after surgery. Expression levels of BCRP were low in comparison with PGP and MRP1 and were not related to relapse or prognosis.

In contrast, a link between PGP gene expression and progression-free survival in advanced disease has been reported in a study of 59 patients with primary operable breast cancer [51]. Patients with no change in tumour size for more than 6 months were defined as having prolonged stable disease. Patients with progressive disease or stable disease with progression within 6 months were classified as nonresponders. The remaining patients were classified as having either a complete or partial response. 22 patients (37%) had a response to treatment, 12 patients (20%) had prolonged stable disease and 25 patients (42%) did not respond to the chemotherapy. The expression of a number of ABC transporters was evaluated in tumour samples from these patients and related to their response to the different chemotherapy regimes. The results can be seen in Table 3 (adapted from [51]). Significant positive correlations between the mRNA levels of drug resistance genes and treatment outcome were seen in particular for PGP/BCRP and PGP/MRP1, in agreement with previous reports [52]. 

Whether MRP1 expression correlates with patient and tumour characteristics was studied in primary breast tumour samples from 259 patients using immunohistochemistry [56]. No significant differences in MRP1 levels were observed according to patient age, menopausal status, tumour size, nodal status or differentiation grade. Cox regression analysis was performed on subgroups of patients stratified by menopausal status, tumour size, nodal status and adjuvant systemic therapy. In node-negative patients with small tumours, MRP1 expression were found to be associated with decreased survival. In node positive patients who received adjuvant systemic chemotherapy, expression of MRP1 was associated with an increased risk of relapse. These data suggest that MRP1 may play a role in chemotherapy resistance in breast cancer, and to be a predictor of poor prognosis in patients that receive first line systemic treatment for recurrence [56]. Although the gene expression levels of PGP in patient samples varied greatly (100-fold), it was still a statistically significant predictor for the type of response to chemotherapy and length of progression-free survival in the cohort of advanced breast cancer patients.

Expression levels of MDR transporters are reported to correlate with disease progression and response to treatment in a study of 104 patients primary invasive breast cancer. High expression levels of PGP as observed by immunohistochemical staining was found to be associated with a higher grade, lymph node involvement, shorter overall survival and a shorter progression-free period [57]. This is supported by a smaller study of 27 breast cancer patients that had all been treated by adjuvant chemo-endocrine therapy after surgery [54]. MRP1 expression was detected in 70% of the breast cancer samples, and was significantly increased compared with normal breast tissue. MRP1 expression levels were higher in tumours of patients that subsequently relapsed compared to those that did not. A study of 50 cases of locally advanced breast cancer relating PGP expression and response to neoadjuvant chemotherapy [53] suggests that PGP expression at diagnosis may predict a poor clinical response to neoadjuvant chemotherapy. A significant correlation was seen between high PGP expression prior to chemotherapy and a poor clinical response. One important finding in this study was that whereas 52% of the patient's tumours were PGP-positive prior to treatment, this increased to 73.5% after treatment, illustrating the limitations of single-time point, pretreatment studies. 

MRP1 expression has been shown to correlate with a shorter relapse-free survival and play a role in resistance to chemotherapy in patients with early breast cancer treated with cyclophosphamide, methotrexate and fluorouracil adjuvant chemotherapy [55]. 1034 patients were stratified by tumour size, number of involved lymph nodes, type of surgery, tumour grade and hormone receptor status, and randomly assigned to receive either six cycles of cyclophosphamide, methorexate and 5-fluorouracil or five years of tamoxifen plus three years of goserelin. MRP1 expression was evaluated using immunohistochemistry in tumour samples obtained at the time of surgery and thus before adjuvant therapy. Expression was categorised and the percentage of patients in each category were similar negative (29% of patients), low (17%), intermediate (25%) and high (29%). There was a weak positive correlation between MRP1 expression and tumour size and grade. MRP1 expression was not significantly correlated with age, lymph node status, hormone receptor status or type of treatment. Univariate analysis demonstrated that younger age, larger tumour size, and a higher number of positive nodes, and increasing levels of MRP1 expression were significantly associated with a shorter relapse-free survival. Large tumour size, higher number of involved lymph nodes, higher tumour grade and higher MRP1 expression were also significantly associated with a shorter overall survival. The independent effects of MRP1 expression on survival were assessed by multiple Cox proportion hazards regression models, taking into account differences in treatment regimes. In the patients treated with cyclophosphamide, methotrexate and 5-fluorouracil, higher MRP1 expression was associated with a shorter recurrence-free survival and overall survival. In contrast, in patients treated with tamoxifen and goserelin, MRP1 expression did not predict recurrence-free survival or overall survival. This patient study indicates that MRP1 expression independently predicts a shorter survival in patients treated with conventional chemotherapy and suggests treatment failure in those patients. 

The contradictory findings reported in a number of clinical studies, may primarily be due to differences in techniques used to assess the levels of MDR pumps. The size and quality of the tumour sample available for study, as well as its composition, is another source of variability. For example, MRP1 mRNA has been detected in 98% of breast cancer samples (containing mixed cell populations) whereas MRP1 protein was detectable in only 53% of the samples [55]. This illustrates that measurements of gene expression alone is likely to lead to an overestimate of the presence of MDR proteins. Alongside the difficulties in detecting the transporters in tumours, intra- and inter-tumour heterogeneity makes reproducible measurements difficult. Often the different proteins are expressed at very low levels, precluding accurate quantification by immunohistochemistry or semi-quantitative RT-PCR [42, 58]. Studies of individual MDR proteins may not be clinically meaningful, as tumours express a whole range of proteins with overlapping functions. Measurements of expression levels of the individual proteins during a course of treatment would increase our understanding of the interactions between the different transporters. Alongside this, information about how therapy affects the functional activity of different ABC transporters would elucidate the role they play in MDR.

Table 4 summarises the findings from key clinical studies investigating the expression of ABC transporters, and illustrates the conflicting results reported regarding the correlation of PGP and MRP1 response to therapy. In general, studies using immunohistochemical methods to detect protein conclude that high tumour levels of PGP and MRP1 indicate shorter survival rates, and an increased risk of relapse. These data suggest that assessing ABC transporter expression levels in breast tumours may help predict patient response to chemotherapy. 

## 10. Conclusions

The exact role of ABC transporters in breast cancer MDR has been difficult to pinpoint due to the complexity of the mechanisms involved. Investigations into the expression of these proteins in breast cancer cells and tumour samples have often proved inconclusive, and differences in the experimental techniques have made it difficult to directly compare results between studies. Although a number of clinical studies have reported that high levels of tumour ABC transporters are associated with tumour progression, a clear link between expression levels and tumour sensitivity to chemotherapy or patient outcome has not been identified. Due to high number of ABC transporters and the redundancy in their function, charting the combined expression levels and functionality may be required to reveal how they interact to generate MDR. Overall, further comprehensive studies are needed to fully elucidate the role that ABC transporters play in breast cancer multidrug resistance. A better understanding of this complex and dynamic system is essential to enable us to develop therapeutic strategies that bypass MDR, and also effective ways of inhibiting MDR components to increase the efficacy of our current extensively used chemotherapies.

## Figures and Tables

**Table 1:** The ABC transporters expressed in breast tissue.

Gene	Protein	Tissue	Chemotherapeutic drugs effluxed by transporter	None chemotherapeutic substrates	References
ABCB1	PGP/MDR1	Intestine, liver, kidney, placenta, blood-brain barrier, most tissues	Colchicine, doxorubicin, etoposide, vinblastine, paclitaxel	Neutral and cationic organic compounds, digoxin, saquinavir, many commonly used drugs	[2, 6–9]
ABCC1	MRP1	All tissues	Doxorubicin, daunorubicin, vincristine, etoposide, colchicines, camptothecins, methotrexate	Glutathione and other conjugates, organic anions, leukotriene C4, rhodamine	[2, 6–9]
ABCC4	MRP4	Prostate, testes, ovary, intestine, pancreas, lung, kidney, most tissues	6-mercaptopurine and 6-thioguanine and metabolites, methotrexate	Nucleotide analoges, organic anions,	[6, 7, 10]
ABCC5	MRP5	Most tissues	6-mercaptopurine and 6-thioguanine and metabolites	Nucleotide analogues, cyclic nucleotides, organic anions	[6, 7, 10]
ABCC10	MRP7	Low in all tissues except pancreas		Nucleoside analogues	[11]
ABCC11	MRP8	Low in all tissues except kidney. Spleen, colon, brain	5-fluorouracil		[7, 8]
ABCC12	MRP9	Breast, testes, brain, skeletal, ovary	Not known	Not known	[8, 12]
ABCG2	BCRP	Liver, breast	Mitoxantrone, topotecan, doxorubicin, daunorubicin, irinotecan, imatinib, methotrexate	Prazosin, pheophorbide A, Hoechst 33342, rhodamine	[6, 13]

**Table 2:** Chemotherapy agents used to treat breast cancer subject to MDR.

Class of drug	Drug	Clinical use	Mechanism of action
Anthracyclines	Doxorubicin	Leukaemias, Hodgkin's Lymphoma, bladder, breast, stomach, lung, ovarian, thyroid, soft tissue sarcomas, multiple myeloma and more	Acts by intercalating DNA, resulting in complex formation which inhibits DNA and RNA synthesis. Triggers DNA cleavage by topoisomerase II resulting in cell death
	Epirubicin	Breast, ovarian, gastric, lung, and lymphomas	Acts by intercalating DNA
Taxanes	Paclitaxel	Ovarian, breast, lung and Kaposi's sarcoma	Mitotic inhibitor; interferes with the normal function of microtubule breakdown. Also induces apoptosis
	Docetaxel	Ovarian, breast and lung	Interferes with microtubule breakdown
Vinca Alkaloids	Vinblastine	Hodgkin's Lymphoma, lung, breast, head and neck and testicular	It binds tubulin, thereby inhibiting the assembly of microtubules
Anti-metabolites	5-Fluorouracil	Breast, head and neck, stomach, colon and some skin cancers	Metabolised to cytotoxic metabolites which are incorporated into DNA and RNA, inducing cell cycle arrest and apoptosis
	Methotrexate	Leukaemia, breast, skin, head and neck and lung	Inhibits metabolism of folic acid. Acts specifically during DNA and RNA synthesis, and thus it is cytotoxic during the S-phase of the cell cycle
Anthracenediones	Mitoxantrone	Breast, Leukaemia, Non-Hodgkin's Lymphoma and Prostate	Topoisomerase II inhibitor; disrupts DNA synthesis and DNA repair

**Table 3:** Effect of ABC transporters on patient response rate to chemotherapy.

	All chemotherapy	Cyclophosphamide. Methotrexate and 5-Fluorouracil	5-Fluorouracil, epirubicin/doxorubicin and cyclophospamide
All patients	34/59 (58%)	15/28 (54%)	19/31 (61%)
Low BCRP	27/42 (64%)	11/20 (55%)	16/22 (73%)
High BCRP	7/17 (41%)	4/8 (50%)	3/9 (33%)
Low MRP1	18/30 (60%)	8/16 (50%)	10/14 (71%)
High MRP1	16/29 (55%)	7/12 (58%)	9/17 (53%)
Low MRP2	18/28 (64%)	5/8 (63%)	13/20 (65%)
High MRP2	13/28 (46%)	8/18 (44%)	5/10 (50%)
Low PGP	32/47 (68%)	13/22 (59%)	19/25 (76%)
High PGP	2/12 (17%)	2/6 (33%)	0/6 (0%)

**Table 4:** Overview of clinical studies investigating the effect of ABC transporters.

Type of Study	No. of patients	Treatment	Detection Method	Outcome	Author
Neoadjuvant	21 patients	5-fluorouracil, epirubicin, cyclophosphamide and paclitaxel	RT-PCR	Differences seen in expression before treatment, no difference in expression response to treatment	[46]
Neoadjuvant	50	Cyclophosphamide, doxorubicin and 5-fluorouracil	Immunohistochemistry	Significant correlation between PGP expression prior to treatment and clinical response	[53]
Adjuvant	171	Chemotherapy +/− radiotherapy +/− hormonal therapy	RT-PCR	No significant correlation between PGP and MRP1 expression and survival	[48]
Adjuvant	85	Anthracycline based	RT-PCR	No significant influence of PGP or MRP1 expression on survival	[49]
Adjuvant	38	Doxorubicin	RT-PCR	No correlation between MRP1 expression and survival	[50]
Adjuvant	27	Chemoendocrine	RT-PCR	High expression levels of MRP1 increased risk of relapse. No significant difference in PGP expression	[54]
Adjuvant	1034	Cyclophosphamide, methotrexate and 5-fluorouracil or tamoxifen and goserelin	Immunohistochemistry	MRP1 expression predicts a shorter survival in patients treated with conventional chemotherapy	[55]
Adjuvant	59	Cyclophosphamide, methotrexate and 5-fluorouracil or 5-fluorouracil, doxorubicin/epirubicin and cyclophosphamide	RT-PCR	High PGP expression significant predictor of poor prognosis	[51]
Adjuvant	259	Cyclophosphamide, methotrexate and 5-fluorouracil	Immunohistochemistry	Increased expression of MRP1 associated with increase in relapse and number of deaths	[56]
Adjuvant	104	Radiotherapy +/− chemotherapy +/− hormonal therapy	Immunohistochemistry	High expression levels of PGP associated with shorter survival	[57]

## Abbreviations

ABC: ATP-binding cassette transporters
ADP: Adenosine diphosphate
ATP: Adenosine triphosphate
BRCA1: Breast cancer 1
BCRP: Breast cancer resistance protein
DNA: Deoxyribonucleic acid
MDR: Multidrug resistance
MRP1: Multidrug resistance associated protein 1
NBD: Nucleotide-binding domain
PGP: P-glycoprotein
RNA: Ribonucleic acid
RT-PCR: Reverse transcription polymerase chain reaction
TMD: Transmembrane domain.
